# Reductive Transformation of ALD TeO_2_ into Continuous and Impurity‐Free Tellurium Films

**DOI:** 10.1002/smll.73470

**Published:** 2026-04-18

**Authors:** Seung Ho Ryu, Seungsu Kim, Taikyu Kim, Jihoon Jeon, Gwang Min Park, Hyeonji Yoo, Sung‐Chul Kim, Sung Ok Won, Ju‐Young Kim, Seong Keun Kim

**Affiliations:** ^1^ Electronic and Hybrid Materials Research Center Korea Institute of Science and Technology Seoul Republic of Korea; ^2^ KU‐KIST Graduate School of Converging Science and Technology Korea University Seoul Republic of Korea; ^3^ Department of Electrical Engineering Stanford University Stanford California USA; ^4^ Department of Materials Science and Engineering UNIST (Ulsan National Institute of Science and Technology) Ulsan Republic of Korea; ^5^ Advanced Analysis and Data Center Korea Institute of Science and Technology Seoul Republic of Korea

**Keywords:** atomic layer deposition, p‐type semiconductors, reductive transformation, tellurium, ultrathin films

## Abstract

The lack of high‐performance p‐type channel materials that can be processed at low temperatures has hindered the progress of monolithic 3D integration. Despite its high hole mobility, Te often exhibits discontinuous island‐like growth when deposited using atomic layer deposition (ALD) due to its weak surface interactions. This study introduces a new reductive transformation method that addresses this inherent issue by converting continuous ALD‐grown TeO_2_ films into crystalline, impurity‐free Te layers. Notably, this approach allows for the formation of fully continuous Te films, even at thicknesses below approximately 5 nm. By utilizing a TeH_2_‐assisted reduction pathway generated in situ, this self‐limiting process ensures complete removal of oxygen from both the bulk and interface regions while preserving exceptional conformality in structures with high aspect ratios. The resulting Te films exhibit excellent electrical properties, such as low contact resistance and stable switching in nonplanar transistor configurations. By decoupling the film continuity from surface wettability, this chemical transformation approach provides a breakthrough solution for integrating ultrathin p‐type chalcogenides into advanced back‐end‐of‐line architectures.

## Graphical abstract

Atomic layer deposition forms a conformal TeO_2_ template that transforms to elemental Te through TeH_2_ mediated reduction using Te(SiMe_3_)_2_ and NH_3_. The transformation is self‐limiting and yields continuous crystalline Te with oxygen‐free bulk and interface even at ultrathin thicknesses. Thin film transistors operate reliably in both planar and nonplanar geometries.

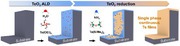

## 1 Introduction

The ongoing miniaturization of Si‐based semiconductor devices is rapidly approaching its physical limits, prompting the search for novel integration strategies. Among these, monolithic three‐dimensional (M3D) integration, in which transistors are stacked in a back‐end‐of‐line (BEOL) structure, has emerged as a promising approach. However, this implementation of complementary‐metal‐oxide‐semiconductor (CMOS) in BEOL M3D integration imposes a stringent process temperature limit below 400°C to prevent degradation of CMOS and interconnects in front‐end‐of‐line structures. This requirement is incompatible with conventional polycrystalline Si channels, necessitating the exploration of new channel materials that can be processed at low temperatures while exhibiting high performance.

Significant advances have been achieved in the development of n‐type channels. Oxide semiconductors, such as In‐Ga‐Zn‐O [1, 2], In─Zn─Sn─O [3, 4], In_2_O_3_ [5, 6, 7, 8], and ZnSnO_x_ [9, 10], as well as transition metal dichalcogenides [11, 12] including MoS_2_, have demonstrated high electron mobility, large on/off ratios, and compatibility with BEOL processing. However, the development of p‐type channel materials has lagged significantly. Although oxide semiconductors such as SnO [13, 14, 15], Cu_2_O [16], and NiO [17], as well as layered chalcogenides [18, 19] including WSe_2_, have been proposed as p‐type channel materials, their performance remains inferior to that of their n‐type counterparts. This imbalance between n‐ and p‐channels impedes the integration of high‐performance and low‐power CMOS devices, suggesting the urgent need for a new p‐type semiconductor.

**FIGURE 1:**
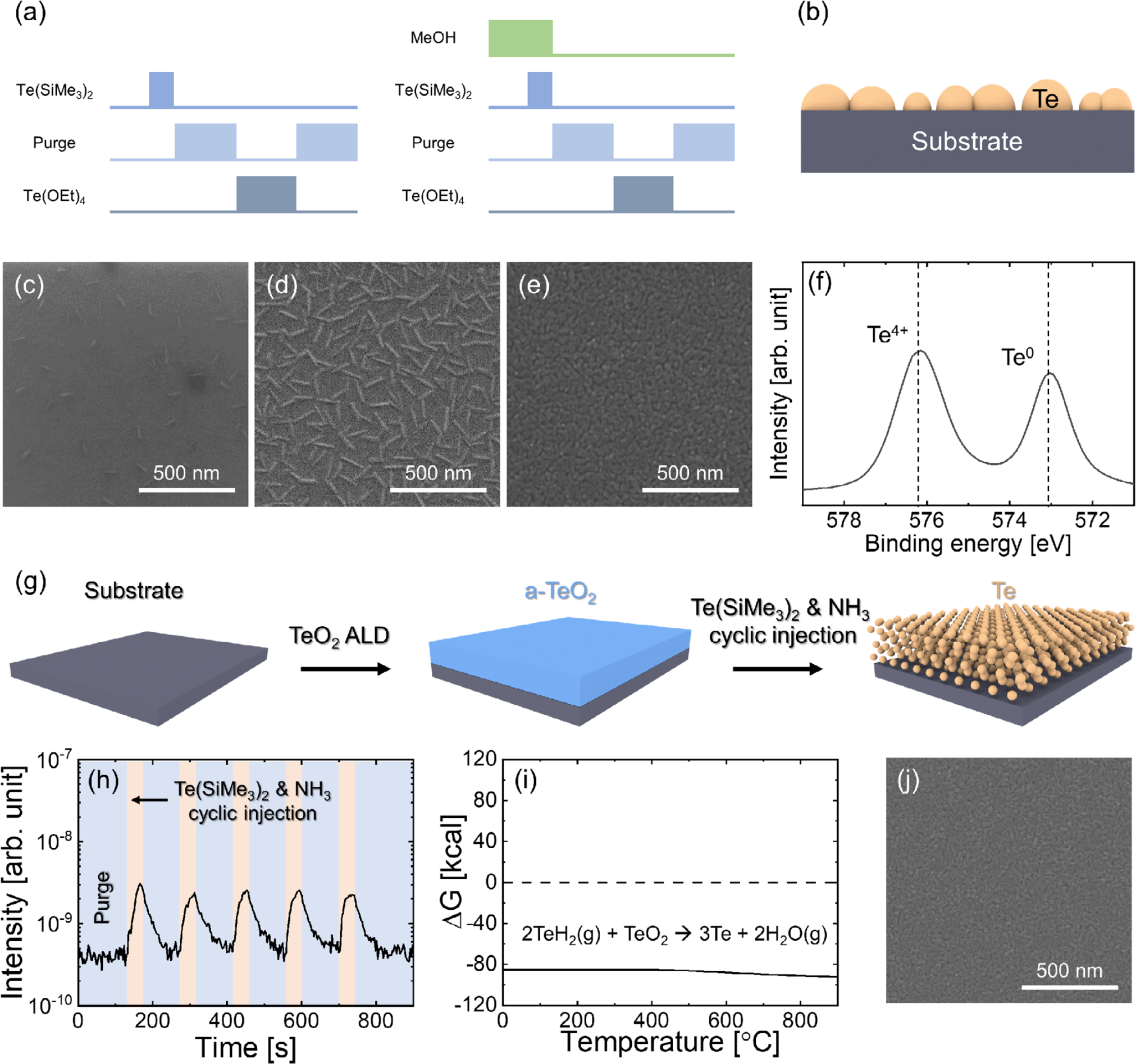
(a) Schematic of two conventional Te ALD recipes. (Left) Common recipe based on ALD reaction between Te(SiMe_3_)_2_ and Te(OEt)_4_. (Right) Modified recipe with co‐injection of MeOH. (b) Schematic of island‐type Te growth caused by poor wettability between Te and the substrate. SEM images of Te deposited on (c) SiO_2_ and (d) ALD‐grown Al_2_O_3_/SiO_2_ using Te(SiMe_3_)_2_ and Te(OEt)_4_, and (e) a Te/TeO_2_ bilayer grown on SiO_2_. (f) Te 3d XPS spectrum of a stacked 4 nm‐thick Te/4 nm‐thick TeO_2_ on SiO_2_. (g) Schematic of the reductive transformation approach for achieving continuous Te films. (h) QMS spectrum of *m/e* = 89 (Me_3_SiNH_2_) during repeated cyclic injections of Te(SiMe_3_)_2_ and NH_3_ followed by purging at 130°C. (i) Calculated Gibbs free energy profile for the reaction between TeO_2_ and TeH_2_ at a temperature range of 0–900°C. (j) SEM image of a 5 nm‐thick Te film produced via transformation of TeO_2_ on SiO_2_.

Recently, Te has garnered significant interest as a promising p‐type semiconductor [20, 21]. Its distinctive electronic structure, characterized by hole pockets near the valence band maximum [22], inherently provides high hole mobility. Moreover, its low melting point enables low‐temperature synthesis, aligning well with BEOL integration. Various synthesis methods, such as sputtering and evaporation, have been employed for its scalable growth [23, 24, 25]. More recently, atomic layer deposition (ALD) has been investigated for Te film growth due to its exceptional uniformity and conformality, crucial for intricate transistor geometries. The ALD surface reaction involving Te(SiMe_3_)_2_ and Te(OEt)_4_ has been reported to produce elemental Te [26], while a modified ALD process incorporating MeOH co‐injection has demonstrated enhanced nucleation (Figure 1a) [27]. Nonetheless, achieving complete surface coverage at ultrathin Te thicknesses through ALD remains a significant challenge. This challenge originates from the intrinsic characteristic of Te, where it comprises 1D helical chains held together by weak van der Waals interactions. The weak interaction between Te chains and typical substrate surfaces leads to poor wettability, promoting island formation and ultimately yielding discontinuous and rough films at the thicknesses necessary for transistor channels (Figure 1b).

Two approaches have been proposed to address these challenges. One approach involves increasing the partial pressure of the Te precursor to enhance nucleation density [28]. However, achieving a uniform distribution of precursors across the entire wafer is challenging, which limits uniformity, and high pressures can lead to poor conformality. Alternatively, using TeO_2_ as an adhesion layer can achieve complete surface coverage [29, 30]. However, this method results in TeO_2_ remaining between Te and the substrate, leading to a degradation in its electrical performance. Consequently, a reliable method to produce conformal, continuous ultrathin Te films without residual oxygen or other impurities has yet to be developed.

In this study, we propose a transformation‐based strategy in which TeO_2_ is initially deposited and subsequently converted into Te. This method facilitates the creation of a continuous ultrathin Te single layer in the absence of oxygen, both within the film bulk and at the interface. Furthermore, excellent conformality was attained. These findings illustrate the potential of this strategy for incorporating BEOL‐compatible Te into emerging electronic devices.

## 2 Results and Discussion

### 2.1 Synthesis of Continuous Te Thin Films

As described in the Introduction, ALD‐grown Te typically proceeds via an island growth mode, failing to form a continuous layer. To confirm this behavior and establish a baseline for comparison with our proposed approach, we analyzed the morphologies of the ALD‐grown Te films. Figure 1c displays a scanning electron microscopy (SEM) image of the surface of Te grown on a SiO_2_ substrate through ALD using Te(SiMe_3_)_2_ and Te(OEt)_4_. Te only partially covered the substrate surface, forming short nanowire‐like crystallites that reflect their intrinsic 1D nature. Despite an increased density of Te grains on the Al_2_O_3_ substrate, which had a relatively high surface energy, full coverage was not achieved (Figure 1d).

As a preliminary study, we also examined the possibility of the approach reported by Kim et al. [29]. and Tan et al. [30], where TeO_2_ was used as a nucleation‐promoting layer for the subsequent Te ALD. As shown in Figure 1e, this approach achieved a continuous Te layer in contrast to the discontinuous films observed in Figure 1c,d. Despite full coverage, the stacked film contained a considerable amount of TeO_2_, as confirmed by X‐ray photoelectron spectroscopy (XPS) spectra shown in Figure 1f. The presence of this TeO_2_ layer resulted in degradation of the electrical performance (Figure S1).

To grow a continuous Te layer without a TeO_2_ interfacial layer, we attempted a reduction strategy, as illustrated in Figure 1g. First, amorphous TeO_2_ films were deposited on a SiO_2_ substrate via ALD using Te(OEt)_4_ and H_2_O at 50°C (Figure S2a). The TeO_2_ ALD process formed single‐phase, smooth and continuous TeO_2_ films (Figure S3). Subsequently, the TeO_2_ films were exposed to cyclic injections of Te(SiMe_3_)_2_ and NH_3_ in a temperature range from 100°C to 150°C (Figure S2b) to transform the TeO_2_ layer into Te. It has been reported that Te(SiMe_3_)_2_ can react with NH_3_ or MeOH to produce TeH_2_ as an intermediate species [27, 31], according to the following reaction:

(1) $$\mathrm{Te}{\left({\mathrm{SiMe}}_{3}\right)}_{2}+2{\mathrm{NH}}_{3}\;\to \;{\mathrm{TeH}}_{2}+2{\mathrm{Me}}_{3}{\mathrm{SiNH}}_{2}$$

The quadrupole mass spectrometer (QMS) spectrum obtained under the cyclic injections of Te(SiMe_3_)_2_ and NH_3_ confirmed the formation of Me_3_SiNH_2_ (Figure 1h), thus verifying the generation of TeH_2_. The expected reaction between TeH_2_ and TeO_2_ is as follows:

(2) $$2{\mathrm{TeH}}_{2}+{\mathrm{TeO}}_{2}\;\to \;3\mathrm{Te}+2H_{2}O$$

This reaction demonstrates a negative Gibbs free energy across the entire temperature range, as illustrated in Figure 1i, indicating the thermodynamic spontaneity of the transformation reaction. Therefore, the overall transformation reaction can be expressed as follows:

(3) $${\mathrm{TeO}}_{2}+2\mathrm{Te}{\left({\mathrm{SiMe}}_{3}\right)}_{2}+2{\mathrm{NH}}_{3}\;\to \;3\mathrm{Te}+2H_{2}O+2{\mathrm{Me}}_{3}{\mathrm{SiNH}}_{2}$$

By reacting the TeO_2_ layers with Te(SiMe_3_)_2_ and NH_3_, continuous Te films with complete surface coverage were successfully formed even at thicknesses as low as approximately 5 nm, as depicted in Figure 1j.

**FIGURE 2:**
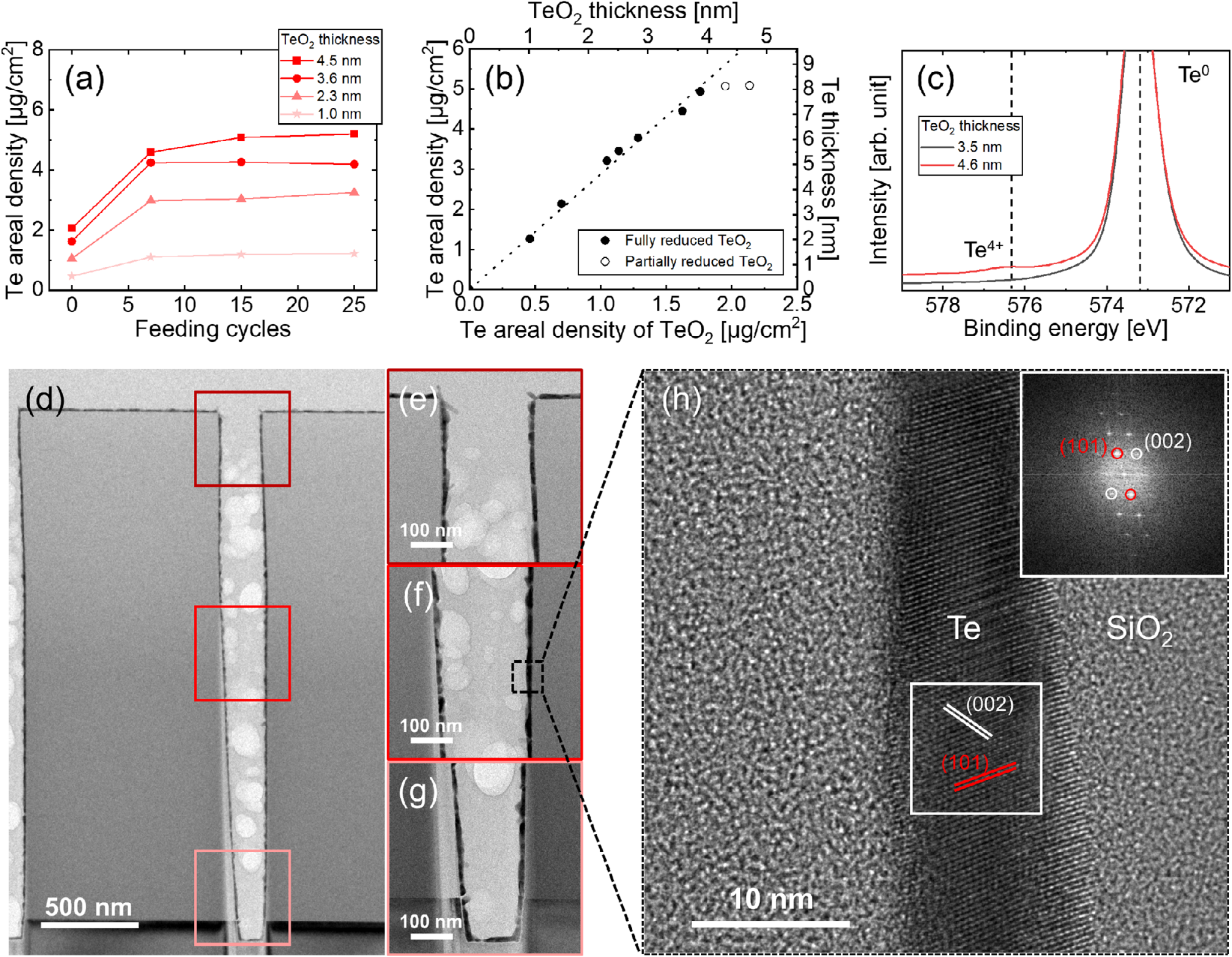
(a) Variation in deposited Te amount for films transformed at 130°C as a function of the number of cycles for TeO_2_ films with different initial thicknesses. (b) Deposited Te amount in the transformed films at saturation as a function of initial TeO_2_ thickness. (c) Te 3d XPS spectra of films transformed from 3.6 nm‐ and 4.7 nm‐thick TeO_2_ films. (d) Cross‐sectional TEM image of a Te film converted from TeO_2_ on a SiO_2_ trench structure with an aspect ratio of ∼12. Magnified TEM images corresponding to the (e) top, (f) middle, and (g) bottom regions of the trench in (d). (h) High‐resolution TEM image of Te in the middle region, with the corresponding fast Fourier transformation pattern shown in the inset.

To comprehend the reaction mechanism during the cycles of alternating Te(SiMe_3_)_2_ and NH_3_ pulses, we investigated the variation in the deposited quantity of Te atoms in the films transformed at 130°C based on the number of cycles for TeO_2_ films with varying initial thicknesses (Figure 2a). The quantity of deposited Te atoms increased with an increasing number of cycles but eventually reached saturation beyond a certain number of cycles. This self‐saturation phenomenon is attributed to the complete consumption of the initially formed TeO_2_ layer, which halts the reaction as depicted in Equation (3). No film growth was detected when the cycles were conducted without the TeO_2_ underlayer (Figure S4), further supporting this interpretation. Figure 2a also illustrates that the deposited Te amount generally increased with the initial TeO_2_ thickness. To further elucidate this trend, we analyzed the change in the amount of deposited Te in the transformed films at saturation concerning the initial TeO_2_ thickness (Figure 2b). The quantity of Te atoms in the transformation‐terminated films increased linearly with the Te content of the initially deposited TeO_2_ films up to a thickness of approximately 4 nm. The slope of the graph is approximately 2.9, indicating that the number of Te atoms in the transformed films is nearly three times greater than that in the initial TeO_2_. This result can be explained by the stoichiometry of Equation (3), where each Te atom in the initial TeO_2_ is accompanied by two additional Te atoms provided by Te(SiMe_3_)_2_ during the transformation, thereby confirming that the transformation progresses in accordance with Equation (3).

However, above an initial TeO_2_ thickness of approximately 4 nm, as depicted in Figure 2b, film growth deviated from linearity and reached saturation. XPS analysis offered additional insights into this phenomenon; the thinner film within the linear regime in Figure 2b comprised solely of Te^0^, while the thicker film beyond this regime showed a slight presence of Te^4+^ (Figure 2c; Figure S5). These findings suggest an incomplete transformation to Te in thicker TeO_2_ films. This incomplete transformation implies a limited reactive penetration depth of TeH_2_, hindering the complete reduction of TeO_2_ layers above a specific thickness.

Therefore, when the thickness of the initially grown TeO_2_ is below this limit, the thickness of the transformed Te is directly determined by the thickness of TeO_2_. This self‐saturation behavior enables the transformation process to enhance uniformity and conformality. To demonstrate this capability, a Te layer was grown using the proposed approach in trenches with a high aspect ratio of approximately 12. Figure 2d shows a cross‐sectional transmission electron microscopy (TEM) image of the grown film. Magnified images of the top, middle, and bottom regions of the trench are shown in Figure 2e–g, respectively, clearly demonstrating conformal film growth across the entire structure. Furthermore, the high‐resolution TEM image in Figure 2h and the inset showing the diffraction pattern of the indicated region prove that the film is continuous and well‐crystallized as Te with no detectable TeO_2_ phase, even at the interface with the SiO_2_ substrate.

**FIGURE 3:**
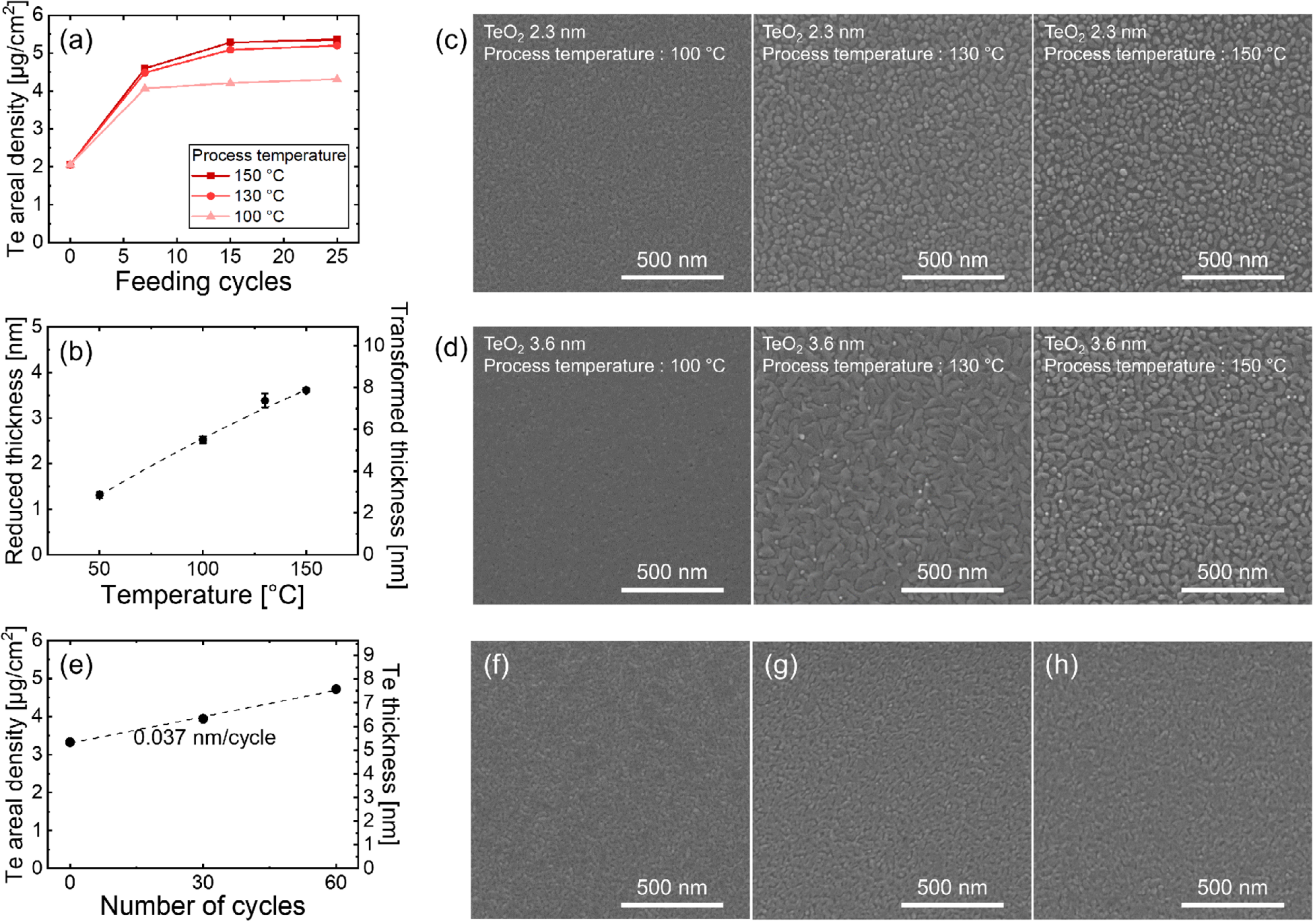
(a) Deposited Te amount as a function of the number of cycles for ∼4.5 nm‐thick TeO_2_ films transformed at 100°C, 130°C, and 150°C. (b) Maximum TeO_2_ thickness that can be fully transformed at each temperature. SEM images of films transformed from (c) 2.3 nm‐ and (d) 3.6 nm‐thick TeO_2_ layers at different transformation temperatures of 100°C, 130°C, and 150°C. (e) Thickness variation of Te films grown by ALD at 50°C on a 5.3 nm‐thick transformed Te layer. SEM images of (f) the initial 5.3 nm‐thick Te layer transformed from TeO_2_, and (g) 6.3 nm‐thick and (h) 7.6 nm‐thick Te films after Te ALD for 30 and 60 cycles, respectively.

The temperature dependence of the transformation with TeH_2_ was also examined. Figure 3a shows the variation in the deposited Te amount of the films as a function of the number of cycles, obtained by transforming approximately 4.5 nm‐thick TeO_2_ films at 100°C, 130°C, and 150°C. Although the initial TeO_2_ thicknesses were identical, the amount of saturated Te increased with the temperature. The maximum TeO_2_ thickness that could be fully transformed at each temperature is displayed in Figure 3b, which clearly shows that the thickness limit increases with the transformation temperature owing to the enhanced reaction kinetics and deeper TeH_2_ penetration.

Once the TeO_2_ thickness is below the limit for a given transformation temperature, complete transformation into Te can be achieved. However, the morphologies of the transformed films were affected by the transformation temperature. Figure 3c,d display the morphologies of films transformed from 2.3‐ and 3.6 nm‐thick TeO_2_ layers, respectively, at different transformation temperatures of 100°C, 130°C, and 150°C. These thicknesses represent the maximum values that could be fully transformed at 100°C and 130°C, respectively. At these maximum transformable thicknesses, the transformed Te films demonstrate full coverage. However, as the transformation temperature rises for the same TeO_2_ thickness, the morphology gradually transitions into island‐like structures. Moreover, even when continuous films are produced, lower transformation temperatures lead to smoother surfaces, while higher temperatures yield rougher morphologies. Additional SEM images illustrating the morphologies concerning temperature, TeO_2_ thickness, and number of cycles are presented (Figures S6–S8).

The temperature‐dependent morphological evolution illustrates a conflict between the completeness of the transformation and film quality. Elevated temperatures accelerated kinetics, facilitating the conversion of thicker TeO_2_ layers but impeding film continuity due to enhanced Te atom mobility. Conversely, lower temperatures resulted in smoother morphologies with complete coverage.

Accordingly, the ability to adjust thickness using the transformation approach is somewhat limited. To address this limitation while preserving the morphology, we introduced an additional Te ALD step after the transformation. In this scheme, the transformed continuous Te layer serves as a seed layer for subsequent Te ALD, allowing further Te deposition while largely preserving the original surface morphology. As shown in Figure 3e, the Te ALD at 50°C on a 5.3 nm‐thick transformed Te layer shows a growth‐per‐cycle of 0.037 nm/cycle. Following deposition, the film thickness increased to 6.3 and 7.6 nm while maintaining a morphology nearly identical to the original transformed layer (Figure 3f–h). These findings illustrate that the proposed transformation method employing Te ALD enables modulation of thickness without compromising morphology.

### 2.2 Absence of Oxygen and Impurities in Bulk and Interfaces

**FIGURE 4:**
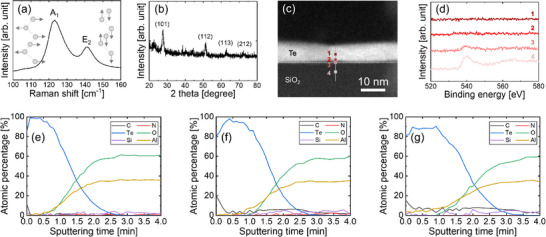
(a) Raman spectrum and (b) grazing‐incident X‐ray diffraction pattern of a Te film obtained by transforming TeO_2_ at 130°C. (c) Scanning TEM image of a Te film transformed at 130°C on SiO_2_. (d) O K‐edge EELS spectra collected at four positions near the Te/SiO_2_ interface: 3.8 nm below the interface, at the interface, and 1 nm and 3 nm above the interface, as indicated in (c). AES depth profiles of the films transformed from TeO_2_ at (e) 100, (f) 130, and (g) 150°C.

As discussed previously, the Te films derived from TeO_2_ exhibited minimal oxygen content, as confirmed by the XPS results presented in Figure 2c and Figure S5. Further analyses were conducted to ascertain the phase purity and, in particular, to verify the absence of oxygen‐related species. The Raman spectrum shown in Figure 4a distinctly illustrates the A_1_ and E_2_ modes at 123 and 140 cm^−1^, respectively, characteristic of trigonal Te. No additional modes were observed. Additionally, the grazing‐incident X‐ray diffraction pattern in Figure 4b exclusively displays Bragg peaks corresponding to Te, devoid of any peaks associated with other phases like TeO_2_. These findings validate the phase purity of the transformed Te films.

In addition to the bulk region, we investigated the interfacial regions with the SiO_2_ substrate, where oxygen could potentially remain even after reduction. Figure 4c shows a scanning TEM image of a Te film transformed at 130°C on SiO_2_. The corresponding electron energy loss spectra (EELS) of the O K‐edge, acquired from four positions near the Te/SiO_2_ interface, are shown in Figure 4d. These spectra were collected 3.8 nm below the interface, precisely at the interface, and 1 nm and 3 nm above the interface, enabling a detailed assessment of oxygen distribution across the boundary. In the spectrum acquired from the SiO_2_ region situated 3.8 nm beneath the interface, only the characteristic peaks of SiO_2_ were observed at energy losses of 540 and 565 eV [32]. The spectrum obtained precisely at the interface also exhibits solely the SiO_2_‐related peak at 540 eV, but with reduced intensity. Notably, the O K‐edge peak corresponding to TeO_2_, typically observed at approximately 537 eV [33, 34], was absent at the interface, confirming the lack of Te‐O bonding. Furthermore, the spectra obtained from the Te regions located 1 and 3 nm above the interface revealed no detectable O K‐edge peaks, confirming that the Te film did not contain oxygen near the interface. These results demonstrate that Te transformed via our proposed strategy has a sharp interface with SiO_2_ that is free of residual oxygen.

The contents of impurities other than oxygen in the transformed films were further examined. Figure 4e–g show the Auger electron spectroscopy (AES) depth profiles of the films transformed from TeO_2_ at (e) 100, (f) 130, and (g) 150°C, respectively. The film transformed at 100°C contained negligible amounts of oxygen, carbon, and nitrogen, which could have originated from the reactants, including Te(SiMe_3_)_2_ and NH_3_. As the transformation temperature increased to 130°C and 150°C, the carbon content slightly increased to ∼2% and ∼5%, respectively, whereas the nitrogen and silicon contents remained nearly constant at ∼0.2 at.% and ∼1 at.%, respectively, across all transformation temperatures. Given that the Si content derived from Te(SiMe_3_)_2_ remained almost constant at all temperatures, the carbon increase was unlikely to be due to thermal decomposition. This is more plausibly attributable to adventitious surface carbon contamination associated with morphology roughening and partial film discontinuity at higher temperatures, rather than being a driving factor for island formation. This interpretation is further supported by the surface detection of Al from the Al_2_O_3_ substrate at 150°C, indicating exposure of the substrate surface due to incomplete film coverage.

### 2.3 Electrical Performances

**FIGURE 5:**
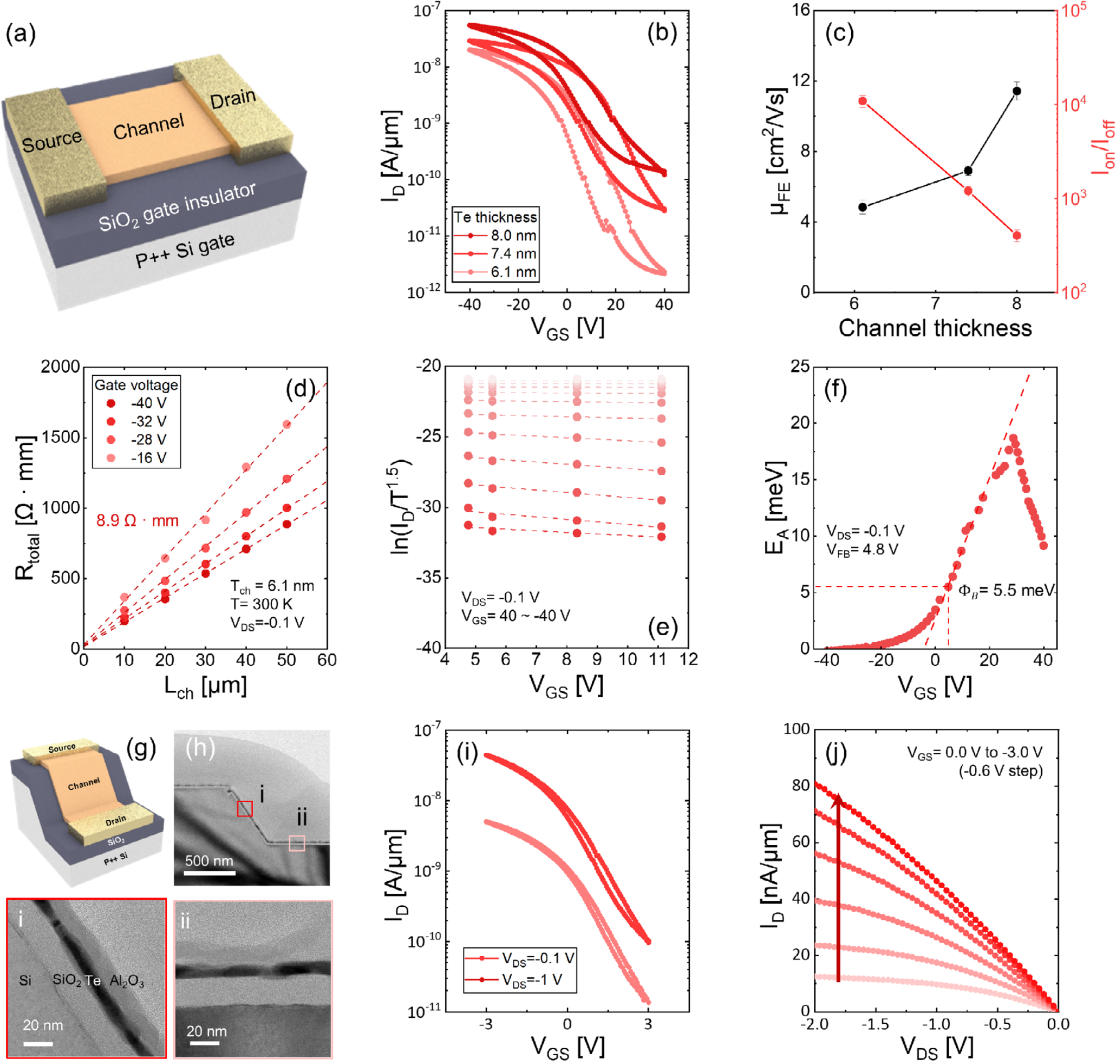
(a) Schematic of the bottom‐gate coplanar TFT structure used to evaluate Te channels formed through the reductive transformation method. (b) Transfer curves for TFTs with 6–8 nm‐thick transformed Te films. (c) µ_FE_ and I_on_/I_off_ extracted from the curves in (b). (d) Variation of the total resistance (R_total_) of the Te TFTs with a 6.1 nm‐thick Te film as a function of channel length. (e) Arrhenius plots of ln(I_d_/T^1.5^) vs. 1000/T at different gate biases used to determine the Schottky barrier height at the Ni/Te interface. (f) Gate‐voltage‐dependent barrier height. (g) Schematic of a nonplanar stepped TFT structure. (h) TEM image of the transformed Te layer formed on a sloped structure with a step height of approximately 600 nm. Magnified TEM images corresponding to the two indicated regions are presented in (h‐i) and (h‐ii). (i) Transfer curves and (j) output characteristics of the non‐planar stepped structured TFT.

To evaluate the electrical performance of the Te films, bottom‐gate coplanar thin‐film transistors (TFTs) were fabricated, as depicted in Figure 5a. These TFTs were passivated with an ALD‐grown Al_2_O_3_ layer. The thickness of the Te channel was varied between 6 and 8 nm to investigate its effect. The transfer curves of the TFTs are presented in Figure 5b. The field‐effect mobility (µ_FE_) and on/off current ratio (I_on_/I_off_) derived from the transfer curves are summarized in Figure 5c. With an increase in Te thickness from 6 nm to 8 nm, the µ_FE_ increased from 4.8 to 11.4 cm^2^/Vs. This trend aligns with findings from previous studies [23, 28, 29, 35]. In contrast, the I_on_/I_off_ ratio decreased from 10^4^ to 4 × 10^2^ as the film thickness increased. This decline can be ascribed to a decrease in the bandgap with increasing film thickness, resulting in elevated off‐state currents [29].

The contact properties between the Te channel and the source/drain electrodes were analyzed using the transmission line method with devices of varying channel lengths. When a gate voltage of –40 V was applied, leading to the accumulation of hole carriers, a contact resistance of 8.9 Ω·cm was determined from the y‐intercept in Figure 5d. This value is comparable to or even lower than those previously reported for Te TFTs [30, 36, 37], indicating efficient carrier injection at the Ni/Te interface. To further elucidate the origin of the low contact resistance, the Schottky barrier height (Φ_B_) between the Ni source/drain and Te channel was determined using the thermionic emission model, which describes the drain current (I_d_) as follows:

(4) $$I_{d}=A^{*}T^{1.5}\exp\left(-\frac{q\Phi_{B}}{kT}\right)\left(1-\frac{qV_{d}}{kT}\right)$$

where *A* ^*^, *T*, *q*, *k*, and *V*_d_ represent the Richardson constant, temperature, electron charge, Boltzmann constant, and drain voltage, respectively. Temperature‐dependent transfer curves were obtained (Figure S9), and the corresponding Arrhenius plots of ln(I_d_/T^1.5^) vs. 1000/T are depicted in Figure 5e. The barrier height dependent on gate voltage was approximated from the slope at each gate voltage, with the Φ_B_ determined to be 5.5 meV under flat‐band conditions (Figure 5f). This minimal barrier height validates the low contact resistance.

To further demonstrate the integration capability of the proposed transformation‐based process, we fabricated TFTs on a nonplanar stepped structure, as shown in Figure 5g. Owing to the excellent conformality of the TeO_2_‐to‐Te transformation, a Te film uniformly covered the sloped structure with a step height of approximately 600 nm, as verified by the TEM images in Figure 5h. The fabricated devices exhibited proper switching behavior even on this nonplanar structure, with an on/off ratio exceeding 10^3^ (Figure 5i) and output characteristics consistent with efficient carrier modulation (Figure 5j). This illustrates that the proposed synthetic approach enables the formation of high‐quality conformal Te channels, underscoring its potential for application in M3D and advanced BEOL device architectures.

## 3 Conclusion

This study reports a reductive transformation strategy that converts ALD‐grown TeO_2_ into continuous, crystalline, and impurity‐free Te thin films, thus addressing the inherent island growth limitation of Te. The transformation occurred through a TeH_2_‐assisted reduction pathway, facilitating self‐limited conversion, exceptional conformality in deep structures, and thorough removal of oxygen from both the bulk and interface regions. The resultant Te channels demonstrate a reasonable µ_FE_, low contact resistance with a Φ_B_ of only 5.5 meV, and consistent switching behavior in planar and non‐planar TFT geometries.

Furthermore, coupling the conversion route with post‐ALD Te deposition allows independent thickness engineering without morphological degradation, thereby offering a scalable route to high‐quality p‐type channels. This study provides a practical platform for BEOL‐compatible Te integration, and transformation chemistry may inspire another pathway for chalcogenide formation. We expect that this transformation‐based approach will facilitate future M3D integration, high‐density logic stacking, and advanced semiconductor device architectures.

## 4 Experimental Section

### 4.1 Film Synthesis

TeO_2_ films were grown on thermally oxidized SiO_2_ (100 nm)/Si substrates in a traveling‐wave type reactor using ALD at 50°C under a working pressure of 0.6 Torr. Te(OEt)_4_ and H_2_O served as the Te and O sources, respectively. A canister containing Te(OEt)_4_ was maintained at 45°C, and the Te(OEt)_4_ vapor was introduced into the chamber with an Ar carrier gas flow of 75 sccm. Another canister holding H_2_O was kept at 18°C, and its high vapor pressure enabled direct delivery into the chamber without a carrier gas. To prevent precursor condensation during delivery, the supply lines were heated to 65°C for Te(OEt)_4_ and 70°C for H_2_O. The pulse step of Te(OEt)_4_ used a cut‐in‐purge scheme, dividing the precursor pulse into two sub‐pulses lasting 2 s each, separated by Ar purging for 5 s. The H_2_O pulse duration was set to 2 s. The precursors were purged with Ar gas for 5 s after the Te(OEt)_4_ pulse and 10 s after the H_2_O pulse to prevent intermixing (Figure S2a).

Cyclic injections of Te(SiMe_3_)_2_ and NH_3_ were performed to reduce the TeO_2_ films. A single reduction cycle consisted of four repetitions of 1 s Te(SiMe_3_)_2_ and 1 s NH_3_ pulses, followed by Ar purging for 10 s (Figure S2b). This cycle was repeated during the reduction. A canister containing Te(SiMe_3_)_2_ was maintained at 25°C, and the Te(SiMe_3_)_2_ vapor was delivered into the chamber with an Ar carrier gas of 75 sccm. The working pressure was approximately 0.6 Torr. The temperature for the reduction process varied in a temperature range from 100°C to 150°C.

### 4.2 Characterization

The amount of Te atoms deposited in the films was examined using wavelength‐dispersive X‐ray fluorescence (ARL Quant'X, Thermo Scientific) with a Rh X‐ray tube target. The surface morphologies of the films were observed by SEM. XPS was employed to examine the chemical binding states of the Te atoms in the films. The phases of the films were verified using Raman spectroscopy and grazing incidence X‐ray diffraction. The impurity content was analyzed by AES. The step coverage and microstructures of the films were investigated using TEM. The elemental composition at the Te/SiO_2_ interface was analyzed using the EELS obtained from STEM. To evaluate the reaction chemistry, QMS experiments were performed by sampling gaseous species through a dedicated line connected to the exhaust line between the reactor and the vaccum pump, which was directly connected to a residual gas analyzer (RGA 200, SRS). For the QMS measurements, Te(SiMe_3_)_2_ and NH_3_ were supplied in a cyclic sequence: one cycle consisted of 20 repetitions of 1 s Te(SiMe_3_)_2_ and 1 s NH_3_ pulses, followed by a 100 s purge step in vacuum (no gas flow). The electrical properties of the TFTs were measured using a semiconductor parameter analyzer (4155 B, Keysight Technology) at room temperature.

### 4.3 Device Fabrication

Planar bottom‐gate TFTs with Te channels were fabricated. A heavily doped p‐type Si substrate was utilized as the gate electrode, and a 100 nm SiO_2_ layer served as the gate dielectric. The Te channel, created through the reduction of TeO_2_ on the gate oxide, was defined using photolithography and subsequently wet‐etched in a 30% H_2_O_2_ solution. Ni (20 nm)/Au (30 nm) bilayer source/drain electrodes were deposited through e‐beam evaporation followed by thermal evaporation. The channel dimensions were 40 µm in width and 20 µm in length.

Nonplanar TFTs were fabricated on an inclined p‐type Si structure formed by anisotropic etching. The anisotropic etching of Si was performed at 70°C in a 45% KOH solution after selectively removing a 100 nm SiO_2_ layer with a 6:1 buffered oxide etchant (BOE; NH4F:HF = 6:1, v/v). Subsequently, a 21 nm‐thick SiO_2_ gate dielectric was deposited through plasma‐enhanced ALD at 100°C using bis‐(diisopropylamino)silane and O_2_ plasma. A Te layer was created by reducing TeO_2_ on the inclined surface, followed by the fabrication of Ni/Au source/drain electrodes on the patterned Te channels. The channel dimensions of the nonplanar TFTs were 28 µm in width and 21 µm in length. All TFTs were passivated with a 10 nm‐thick Al_2_O_3_ layer deposited by ALD at 120°C using trimethylaluminum and H_2_O.

## Conflicts of Interest

The authors declare no conflicts of interest.

## Supporting information




**Supporting File**: smll73470‐sup‐0001‐SuppMat.pdf.

## Data Availability

The data that support the findings of this study are available from the corresponding author upon reasonable request.
