# Supplementary material for: Reductive Transformation of ALD TeO2 into Continuous and Impurity‐Free Tellurium Films
Source: Small. 2026 Apr 18;22(32):e73470. doi: 10.1002/smll.73470 (PMC13244410; doi:10.1002/smll.73470)
Supplement: Supplementary file 1 — Supporting File: smll73470‐sup‐0001‐SuppMat.pdf. [file SMLL-22-e73470-s001.pdf]

Supporting Information

Reductive Transformation of ALD TeO_2 into Continuous and Impurity-Free Tellurium Films

*Seung Ho Ryu, Seungsu Kim, Taikyu Kim, Jihoon Jeon, Gwang Min Park, Hyeonji Yoo, Sung-Chul Kim, Sung Ok Won, Ju-Young Kim, Seong Keun Kim**

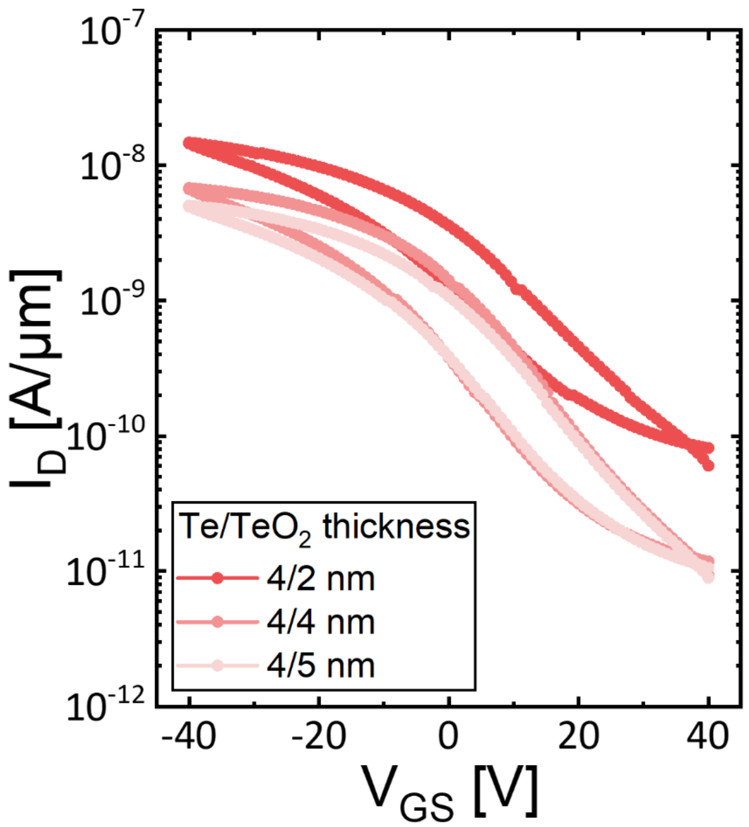

Figure S1. Transfer curves of TFTs with 4 nm-thick ALD Te films deposited on TeO_2 layers with initial thicknesses of 2, 4, and 5 nm.

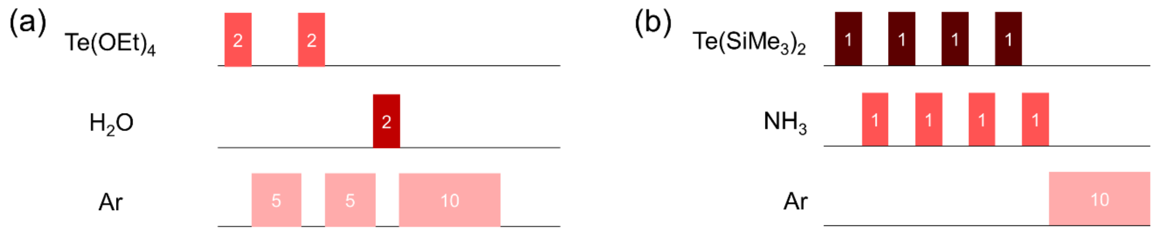

Figure S2. Schematics of (a) the ALD process used to deposit TeO_2 films and (b) the cyclic injection process used to transform TeO_2 into Te.

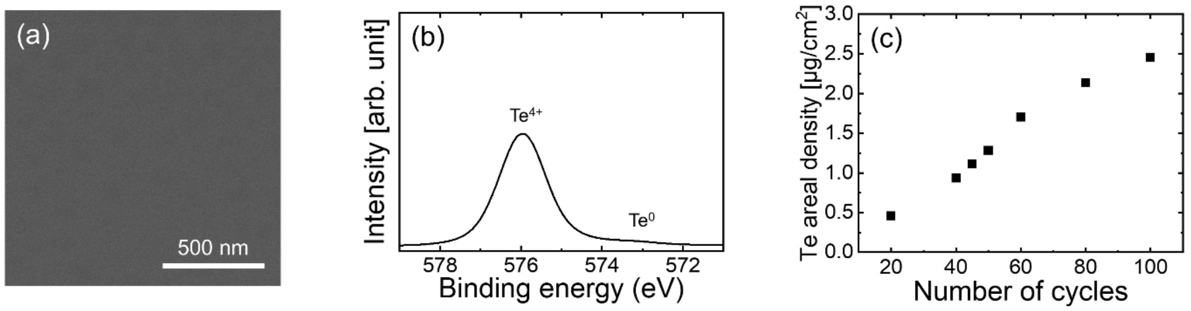

Figure S3. (a) SEM image of an amorphous TeO_2 film deposited for 100 ALD cycles. (b) Te 3d XPS spectrum of TeO_2 deposited at 50 °C. (c) Variation in Te areal density as a function of the number of ALD cycles.

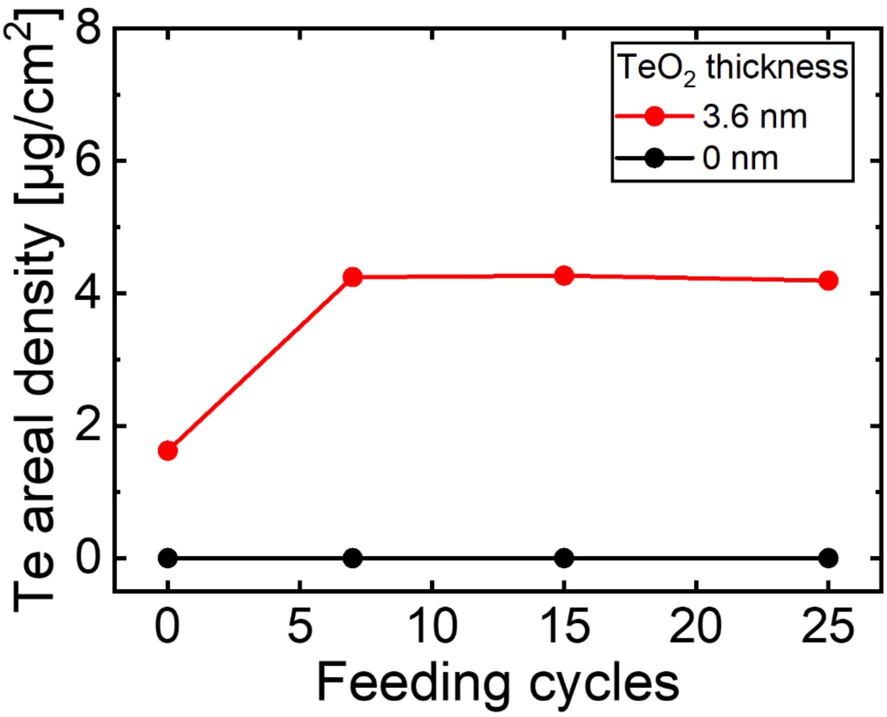

Figure S4. Variation in Te areal density during cyclic injection of $Te(SiMe_3)_2$ and NH_3 at 130 °C for samples with and without a 3.6 nm-thick TeO_2 film.

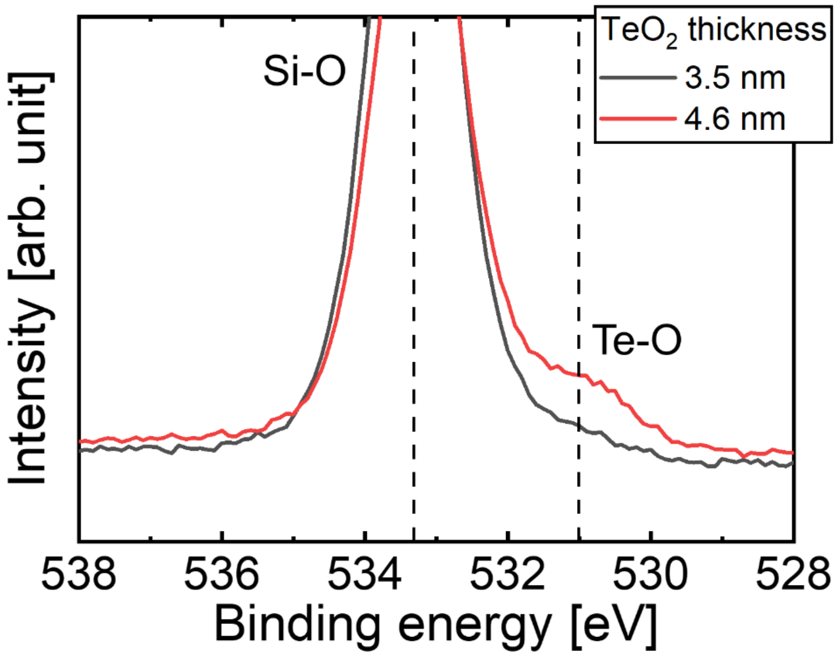

Figure S5. O 1S XPS spectra of films transformed from 3.6 nm- and 4.7 nm-thick TeO_2 films.

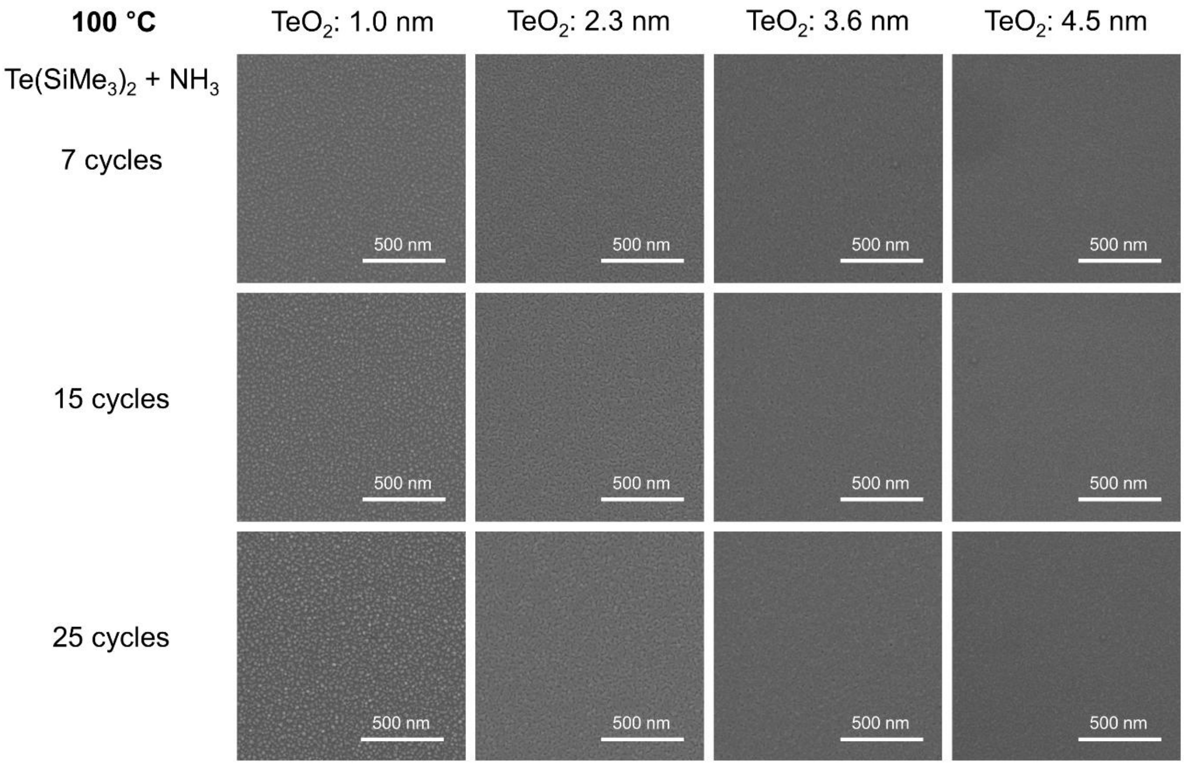

Figure S6. SEM images of Te films transformed from TeO_2 layers of different initial thicknesses after varying numbers of $Te(SiMe_3)_2/NH_3$ cyclic injections at 100 °C.

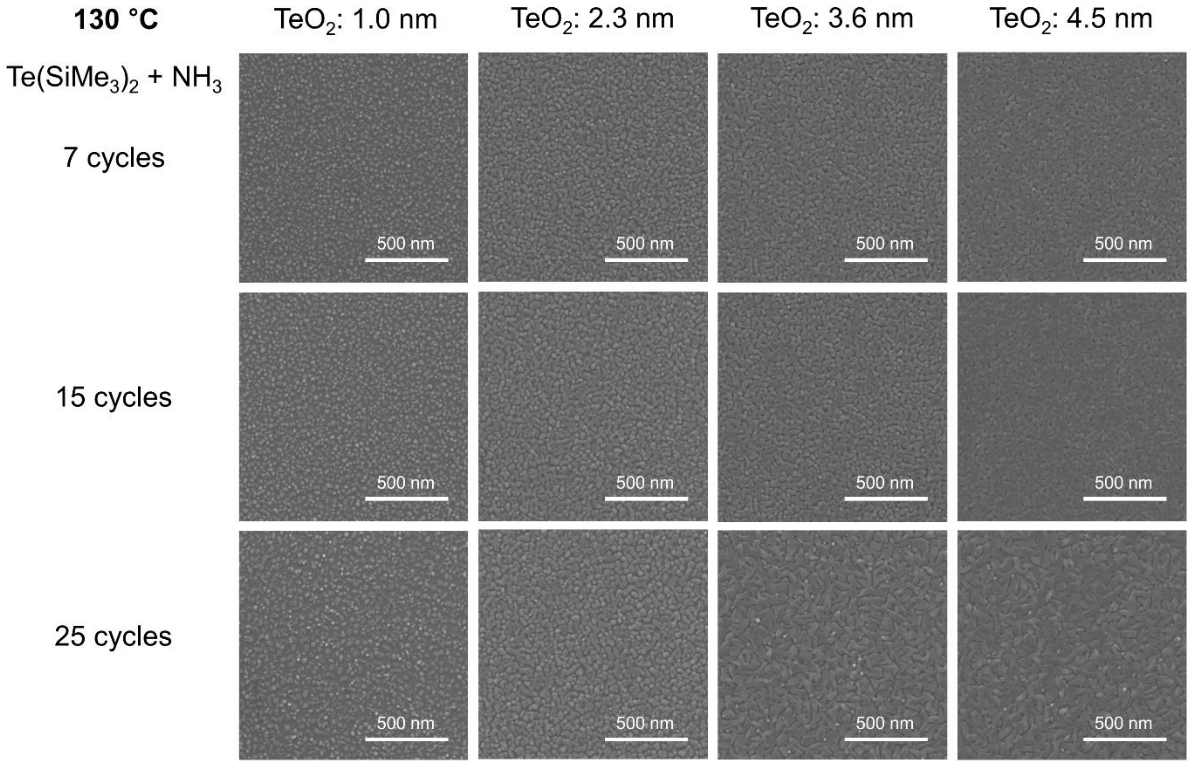

Figure S7. SEM images of Te films transformed from TeO_2 layers of different initial thicknesses after varying numbers of $Te(SiMe_3)_2/NH_3$ cyclic injections at 130 °C.

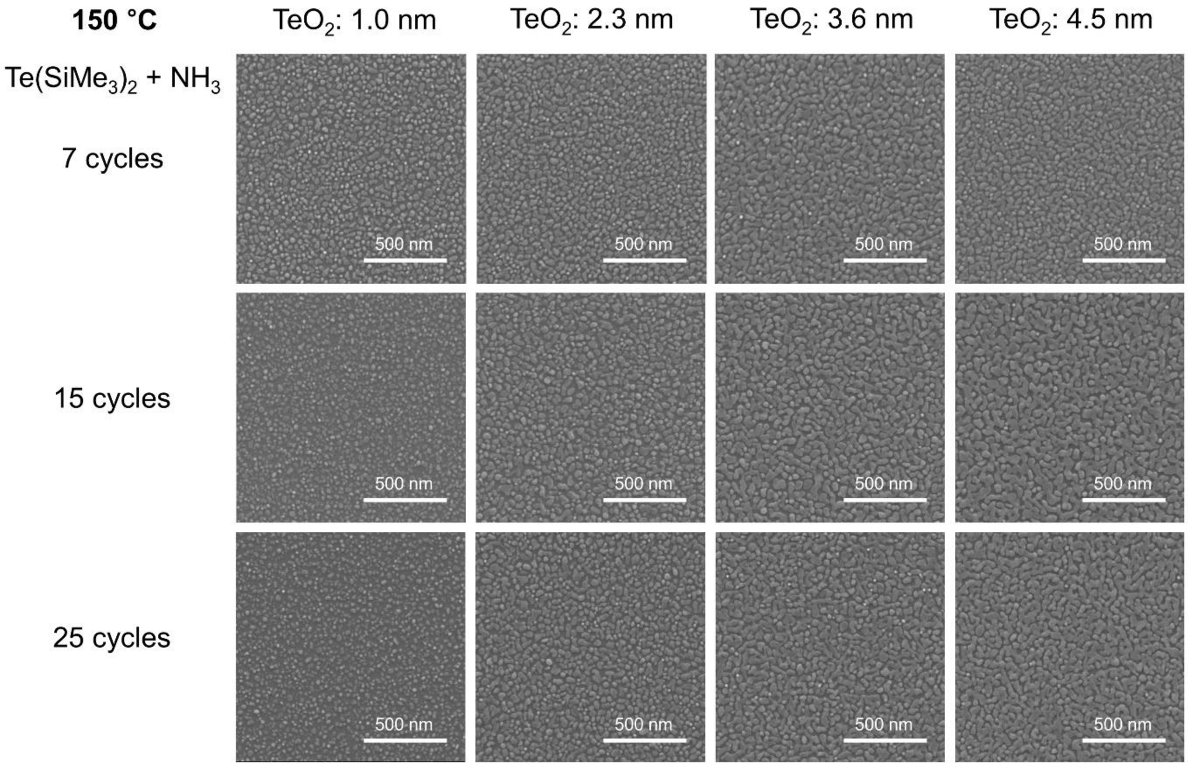

Figure S8. SEM images of Te films transformed from TeO_2 layers of different initial thicknesses after varying numbers of $Te(SiMe_3)_2/NH_3$ cyclic injections at 150 °C.

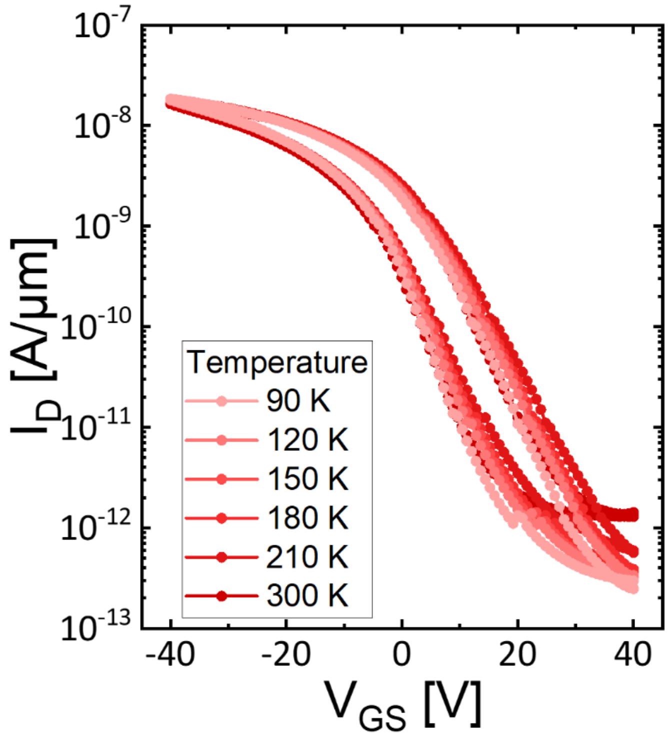

Figure S9. Temperature-dependent transfer curves of TFTs incorporating 6.1 nm-thick Te films.
